# Enhancing brain tumor segmentation in MRI images using the IC-net algorithm framework

**DOI:** 10.1038/s41598-024-66314-4

**Published:** 2024-07-08

**Authors:** Chandra Sekaran D S, J. Christopher Clement

**Affiliations:** School of Electronics Engineering, Vellore Institute of Technology, Vellore, Tamilnadu 632014 India

**Keywords:** Biomedical engineering, Medical imaging

## Abstract

Brain tumors, often referred to as intracranial tumors, are abnormal tissue masses that arise from rapidly multiplying cells. During medical imaging, it is essential to separate brain tumors from healthy tissue. The goal of this paper is to improve the accuracy of separating tumorous regions from healthy tissues in medical imaging, specifically for brain tumors in MRI images which is difficult in the field of medical image analysis. In our research work, we propose IC-Net (Inverted-C), a novel semantic segmentation architecture that combines elements from various models to provide effective and precise results. The architecture includes Multi-Attention (MA) blocks, Feature Concatenation Networks (FCN), Attention-blocks which performs crucial tasks in improving brain tumor segmentation. MA-block aggregates multi-attention features to adapt to different tumor sizes and shapes. Attention-block is focusing on key regions, resulting in more effective segmentation in complex images. FCN-block captures diverse features, making the model more robust to various characteristics of brain tumor images. Our proposed architecture is used to accelerate the training process and also to address the challenges posed by the diverse nature of brain tumor images, ultimately leads to potentially improved segmentation performance. IC-Net significantly outperforms the typical U-Net architecture and other contemporary effective segmentation techniques. On the BraTS 2020 dataset, our IC-Net design obtained notable outcomes in Accuracy, Loss, Specificity, Sensitivity as 99.65, 0.0159, 99.44, 99.86 and DSC (core, whole, and enhancing tumors as 0.998717, 0.888930, 0.866183) respectively.

## Introduction

Segmenting brain tumors is a crucial step in neuro-oncology that helps with accurate diagnosis, therapy planning, and therapeutic oversight. In this study, we developed the IC-Net (Inverted-C) architecture to enhance the segmentation of brain tumors from medical imaging data. The special issues raised by tumor heterogeneity, irregular forms, and the demand for greater accuracy are addressed in our proposed research work. Due to the enormous quantity of information that Magnetic Resonance Imaging (MRI) provides as well as the variability in the position and scope of the tumor, computerized segmentation is a difficult process^1^. Therefore, to accurately segregate the tumorous region from healthy tissues of our human brain. By accurately predicting both the core and peritumoral regions of brain tumors, the IC-Net design seeks to achieve higher segmentation accuracy. This accuracy is essential for planning treatments and subsequent evaluations of treatment effectiveness, allowing doctors to customize therapies and improve patient care.

Brain tumor segmentation (BTS) is crucial for various reasons, including treatment planning, monitoring disease progression, conducting clinical trials, providing measurable data for medical research, extracting of meaningful features and aiding in image-guided navigation^2^. It allows for precise definition of tumor boundaries, enabling targeted therapies like surgical resection, radiation therapy, or chemotherapy. It also aids in tracking changes in tumor size and shape over time, allowing for a better understanding of the tumor’s response to therapy and disease development. Additionally, it aids in extracting meaningful features from the segmented regions, such as physical traits, texture, and intensity, for a more comprehensive tumor investigation.

For the purpose of performing image segmentation tasks in the area of medical imaging, specifically to provide masks to segment images for brain tumors if found in brain MRI scans, we have investigated novel convolution neural network (CNN)-based model, named IC-Net. Developed models were created using U-Net as their foundation. The recently put forth models have made use of the Merge Block concept to incorporate together the global and local context, as well as the Attention Block concept to concentrate on the region of interest that contains a certain object.

Due to its effectiveness and efficiency, the U-Net architecture has been widely acknowledged and used in medical image segmentation applications, including BTS^3^. To precisely adapt the conventional U-Net design to the subtleties and difficulties of BTS, we have made several changes to it. Our IC-Net model tackles the challenge of precise BTS, discussing tumor size, form, and variation, overlapping structures, the adjustments improve the network’s capacity to precisely, and emphasizing on locating erratic tumor boundaries in MRI scans of the brain. Addressing the enormous data for training and deploying models on real-time implementations and also focus on uneven border variation within the brain’s tumor in MRI is a significant challenge.

The standard procedure for analyzing brain tumors at the moment is to use Computer Tomography (CT) or MRI imaging to assess the pathological status of brain tissue. The benefits of various imaging methods for tumor diagnosis vary^4^. In contrast to CT imaging, Hussain, Shah, et al. have reviewed and discussed a few techniques that employ MRI and noninvasive imaging techniques that can give the viewer high-quality images free of damage and skull artefacts, with a clear understanding of the anatomical structure, and with excellent soft tissue resolution^5^. By altering the pertinent settings, intracranial pictures in any direction can be produced concurrently. Moreover, MRI of the same tissue at various angles or utilizing various modalities can be produced using various imaging sequences. A multimodal MRI image is the common name for this kind of imaging^6^. Multimodal MRI scans are samples of the same tissue at various contrast levels, acquired using several MR development techniques^7^. Young Kim, Eun, and Hans J. Johnson discussed about water molecules that are present in brain tissue devoid of tumors and other lesions start to experience lesion effects, such as tissue edema^8^. The water molecules in the bonded state are visible as high target in Flair and T2 data. Hence, it is potentially possible to segment the entire tumor using Flair MRI images as the primary foundation. Nonetheless, the tumor will also exhibit asymmetrical alterations in the Flair image due to several unique situations^9^.

While executing the training, attention mechanism-based U-Net models constantly highlight the pertinent activation’s, which results in a superior generalization of the networks. By doing this, we can cut down on the amount of CPU power needed to process pointless activation’s. Hard attention and soft attention are the two different categories of attention systems. The hard attention method performs the picture segmentation operation by cropping the image to highlight the relevant parts. Because it uses a non-differentiable strategy and only works on one area at a time, this type of attention mechanism requires reinforcement learning in order to be back-propagated. In contrast, the soft attention mechanism employs a weighting strategy to give higher weights to the pertinent elements containing the regions of interest and lower weights to the non-relevant parts, which primarily make up the background area of the image. Section "Introduction" provides a thorough introduction to brain tumor followed by a clear explanation of related work in section "Related works". Section "Dataset" provides a brief description about the datasets. In section "Pre-processing", pre-processing where the different processing method are explained. Section "Methodology" Detailed Methodology of our IC-Net model is stated. In section "Results and discussion", we present our results which have been explained. We conclude our work by section "Conclusion".

The following characteristics of the intended models are crucial:

1. To reduce computational complexity, IC-Net, a novel end-to-end framework for brain tumor segmentation, uses fewer convolutional layering techniques for both up- and down-convolution operations.
2. Using multiple convolutional processes, the MA-Block (Multi-Attention) function enhances feature representations and captures intricate patterns, whereas IC-Net combines global features and contextual data.
3. To ensure that the network’s attention focuses on key image areas, the Attention block is employed during segmentation to concentrate on particular areas of interest, such as brain tumors.
4. The suggested model works better than most DL-based models by producing better results with a reasonable amount of sample labels and the right kinds of data augmentation techniques.

### Contributions of IC Net architecture

Convolutional layers for multilevel feature learning, batch normalization along with activation stabilization, selective feature importance, extraction of heterogeneous data, adaptive feature integration, and improved model representation make up the IC-Net framework. These functionalities enhance the management of complex information representations, draw attention to significant characteristics, and boost productivity for operations like feature extraction and segmentation. The FCN makes it easier to extract different feature representations and data types, and the attention mechanism aids in keeping the model focused on pertinent areas.

## Related works

This section discusses various BTS methods, including traditional and Deep Learning (DL) models like U-Net, FCN, and attention-based networks, and their limitations, emphasizing the need for developed network architecture IC-Net.

Modern medical image segmentation methods like U-Net make use of the idea of an encoder-decoder framework^10^. These segmentation models combine the low-level, fine-grained aspects of the encoder, or contracting path, with the high-level, coarse-grained attributes of the decoder, or expansive path, using the idea of skip connections. When doing medical image segmentation tasks, this concatenation is useful for producing reconstructed fine-grained characteristics of the target segmented mask. A U-shaped design results from the symmetrical construction of both the contracting and expansive paths. On the other hand, because the characteristics captured by those down-sampling layers within the ’contracting path’ are weak and correlate to a lot of lower-level features, the restoration of spatial information via these concatenations could fail to deliver precise information. We highlight the pertinent segments of the segmentation masks because such lower-level features could improve the irrelevant regions of the object being targeted segmentation masks.

Over the years, numerous approaches have been proposed for BTS, and CNNs have emerged as a powerful tool in this domain. The U-Net architecture, introduced by Ronneberger et al. has outperformed and widespread attention for its exceptional performance in biomedical image segmentation tasks, including BTS^11^. According to the study, which tests networks with higher levels of depth using small (3$\times$3) convolution filters, improving networks to 16-19 weight layers could result in a significant improvement^12^. In the study, the Edge U-Net model is used, which is a deep CNN that can accurately find the location of tumors by combining boundary-related MRI data with brain MRIs and a loss function^13^. The study was illustrated automatic BTS using hybrid filters and 3D medical pictures. The U-Net model is used for semantic segmentation, and 2D MRIs are used to view the tumor. The method is computationally and memory-efficient, proving to be the optimal choice for segmenting brain images^14^.

Baid, U. et al were designed a method for glioma tumor segmentation and survival prediction using a Deep Learning Radiomics Algorithm for Gliomas (DRAG) model and a 3D patch-based U-Net model. The model achieved 57.1% accuracy on the BraTS 2018 validation dataset, performing well and achieving the overall survival prediction task^15^. The model uses the MICCAI BRATS2018 to classify tumor types in MRI images based on their masses. It achieves significant results in segmenting tumors using DL approaches, with dice coefficient values for high-grade glioma volumes and low-grade glioma volumes of 0.9795 and 0.9950 respectively^16^. The research has examined an SPP-U-Net, a model that replaces residual connections with Spatial Pyramid Pooling and Attention blocks, enhancing reconstruction scope and context. It achieves comparable results without changing parameters over larger dimensions^17^. Sunita Roy, et al. proposed two new CNN-based models, S-Net and SA-Net, for image segmentation in medical imaging, particularly for brain tumors in MRI scans. These models use U-Net as the base architecture and leverage ’Merge Block’ and ’Attention Block’ concepts^18^.

Ruba,T, et al proposed a JGate-AttResU-Net network design for a reliable BTS system, enhancing tumor localization and generating competitive outcomes using the BRATS 2015 and 2019 datasets^19,20^. BrainNET is a new network that uses DL networks to automate the detection and classification of brain tumors from MRI images, overcoming the complexity and variance of tumors and medical data in clinical routines^21^. Yanjun Peng and Jindong Sun suggested an automatic weighted dilated convolutional network (AD-Net) for learning multimodal brain tumor features through channel feature separation learning. The AD unit uses dual-scale convolutional feature maps, two learnable parameters, and deep supervision training to quickly fit the data using the BraTS2020 dataset^22^. ^23^TransBTS validates the efficiency of a Transformer-based framework for visual tracking, emphasizing attention mechanisms for healthy feature learning.

Rehan Raza, et al. introduced a 3D BTS framework, a hybrid of deep residual networks and U-Net models, that significantly enhances the segmentation performance of brain tumor sub-regions when compared to state-of-the-art techniques^24^. The 2D U-Net network, trained on the BraTS datasets, can identify four areas for BTS. It can set up multiple encoder and decoder routes for various image usages. Image segmentation is used to reduce computational time. Experiments on the BraTS datasets demonstrate the model’s effectiveness^25^. This study presented a fully automated 2D U-net architecture on the BraTS2020 for detecting tumor regions in healthy tissue. After experimenting with all MRI sequences, the model achieved an accuracy of 99.41%, demonstrating its effectiveness. The model is further trained to assess its robustness and performance consistency^26^. N. Phani Bindu and P. Narahari Sastry, improve accuracy, cross- or skip-connections between network blocks can be introduced. This approach improves model accuracy and performance compared to traditional U-Net models, as it eliminates the need for frequent skip connections^27^.

**Table 1:** Comparative analysis with other pertinent strategies using the BRATS dataset.

Attention mechanism	Features	Advantages	Effectiveness in segmentation tasks
3D Attention U-Net^28^	Convolution-based recalibration	Segmentation accuracy improvement	High
Patch-based 3D UNet with Attention^29^	Selective feature recalibration	Enhanced delineation of relevant regions	Moderate
Attention with Skip Connections^30^	Optimized feature extraction	Context-based feature enhancement	Significant
Large-Kernel Attention^31^	Global context enhancement	Understanding complex structures	Substantial
Self-Calibrated Attention U-Net^32^	Adaptive recalibration	Handling complex structures	High
Dual-Attention Mechanism^33^	Dual attention for intricate features	Precise delineation	Considerable
Attention-Guided CNN^34^	Dynamic feature extraction, multi-scale context.	Improves spatial feature representation, reduces computational cost.	Considerable
Fully Automated Multimodal^35^	Dual attention for intricate features.	Focus on tumor regions, suppress irrelevant background	High

The study employs a model for medical image semantic segmentation using CNNs. The model eliminates semantic ambiguity in skip connection operations by adding attention gates, combining local features with global dependencies, and using multi-scale predictive fusion^36^. A modified U-Net structure using residual networks and sub-pixel convolution was proposed, enhancing modelling capability and avoiding de-convolution overlapping. The model was evaluated on the BTS dataset, achieving accuracies of 93.40% and 92.20% and outperforming existing approaches in tumor subregion classification^37^. Amin, Javeria, et al proposed model uses 07 layers for classification, including convolutional, ReLU, and softmax layers. It divides MR images into patches and assigns labels. Experiments were conducted on eight large-scale datasets, and results were validated on accuracy, sensitivity, specificity, precision, and similarity index^38^. Raut, Gajendra, et al. classify new input images as tumorous or normal, use back propagation for accuracy and autoencoders for irrelevant features. The K-Means algorithm is used for further tumor region segmentation^39^.

The study uses the 3D-U-Net model for volumetric segmentation of MRI images and tumor classification using CNNs. Validity is established through loss and precision diagrams. Performance is measured and compared, finding the proposed work more efficacious than state-of-the-art techniques^40^. Agrawal P, Katal N, Hooda N. introduce a fully automatic method for separating brain tumours using U-Net-based deep convolutional networks. This method was tested on BRATS 2015 datasets with 220 high-grade tumour cases and 54 low-grade tumour cases, and cross-validation showed that it worked well for separating tumours^41^. This paper presents a DL method for segmenting brain tumours into subregions using a multitask framework and a three-stage cascaded framework for simultaneous and sequential segmentation^42^.

The study explores an attention-based U-Net for brain tumor MRI scans. The model uses U-Nets to segment glioma subregions using T1, T2, T1CE, and FLAIR modalities. The model segmented WT, TC, and ET using the FLAIR modality, achieving scores of 95.56, 93.31, and 89.95 over BraTS 2018^28^. Feng, Xue, et al. proposed a patch-based 3D UNet with an attention block. Findings are mean Dice scores of 0.806 (ET), 0.863 (TC), and 0.918 (WT) in the validation dataset^29^. Na Li and Kai Ren developed DAU-Net, an attention-based nested segmentation network. A deep supervised encoder-decoder architecture and a redesigned dense skip connection to identify key feature regions and merge extracted features^33^. A large-kernel (LK) attention module that integrates convolution, self-attention, and channel adaptation was proposed by Li, Hao, et al. for efficient multi-organ and tumor segmentation^31^.

SCAU-Net is a 3D U-Net model for brain tumor segmentation, employing external attention and self-calibrated convolution modules. It achieves competitive results on the BraTS 2020, 2018 and 2019 validation datasets^32^. In this work, a 3D U-Net model that uses different skip connections with preset 3D MobileNetV2 and attention blocks has been developed by Chinnam SK, Sistla V, and Kolli VK by implementing 3D brain imaging data^30^. Comparative analysis with other pertinent strategies using the BRATS dataset are stated in Table 1. Advanced optimization techniques like stochastic gradient descent and adaptive learning rate algorithms are being used to improve the performance of modified U-Net models for BTS.The integration of attention mechanisms, residual blocks, and advanced optimization techniques has led to significant improvements in segmentation accuracy and performance.

We recognize that our proposed blocks leverage combinations of existing methodologies, such as MA-Blocks, FCN blocks, and attention mechanisms. However, the true innovation of our work lies in the specific integration and optimization of these blocks within a unified framework tailored for the segmentation of brain tumors. Our IC-Net framework strategically combines these techniques to enhance feature extraction, improve segmentation accuracy, and address specific challenges in medical image analysis that individual blocks alone might not solve as effectively. This integrated approach results in a synergistic effect that significantly improves the performance metrics, as demonstrated in our comprehensive experimental results. We believe that this novel integration and its application to the specific domain of BTS contribute meaningful advancements to the field, providing a robust and effective tool for clinical use.

## Dataset

**Figure 1:**
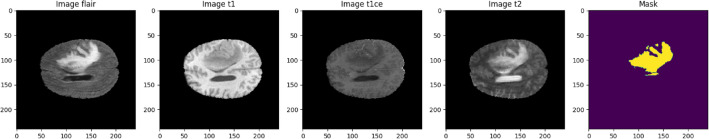
MRI sequences as 2D sections.

The benchmark datasets used in this study are covered in this section’s discussion. The BraTS2020 dataset, obtained from Kaggle, serves as the basis for our system’s analysis and training. Four MRI sequences are collected for each patient: fluid attenuated inversion recovery (FLAIR), T1-contrast-enhanced (T1ce), T1-weighted (T1), and T2-weighted (T2), along with the associated data sample shown in Fig. 1. The training brains come with ground truth for which 5 segmentation labels are provided, namely non-tumor, necrosis, edema, non-enhancing tumor and enhancing tumor. The experts assigned labels to the presented ground truths. Each 3D volume has 155 2D slices/images of brain MRIs that were gathered from different parts of the brain. All brains in the dataset have the same orientation. In NIfTI format, each slice has a size of $240\times 240$ pixels and is composed of single-channel grayscale pixels. Table 2 provides a summary of the dataset.

**Table 2:** Summary of the dataset.

Task	Description
Dataset	BraTS2020
Dataset source	Kaggle
Image type & format	3D brain MRI & NIfTI
Image size	224$\times$224$\times$150
Total number of subjects and No. of images in each subject	473 and 5
Name of the modalities	FLAIR, T1, T1ce, T2 and Seg
Segment Classes	Non-tumor, necrosis, edema, non-enhancing and enhancing tumor

## Pre-processing

Preprocessing methods get the medical images ready for segmentation task training on a DL model are discussed. In order for the model to gain knowledge from the data during training, it must be in an appropriate format and contain relevant information. Data pre-processing is required to eliminate noisy regions and extract crucial segmentation properties before importing the dataset into the training model, ensuring clear labeling. The nib library is used for loading medical image data from NIfTI files, while data shuffling and data resizing are used for training models. Class mapping and One-Hot Encoding are used for segmentation tasks. Normalization ensures pixel values fall within a similar range, while data yielding generates batches of preprocessed data for training and evaluation.

## Methodology

In this manuscript, we introduce IC-Net model that incorporates MA-blocks, attention blocks and FCN block. Our contributions can be summarized as follows:

**Figure 2:**
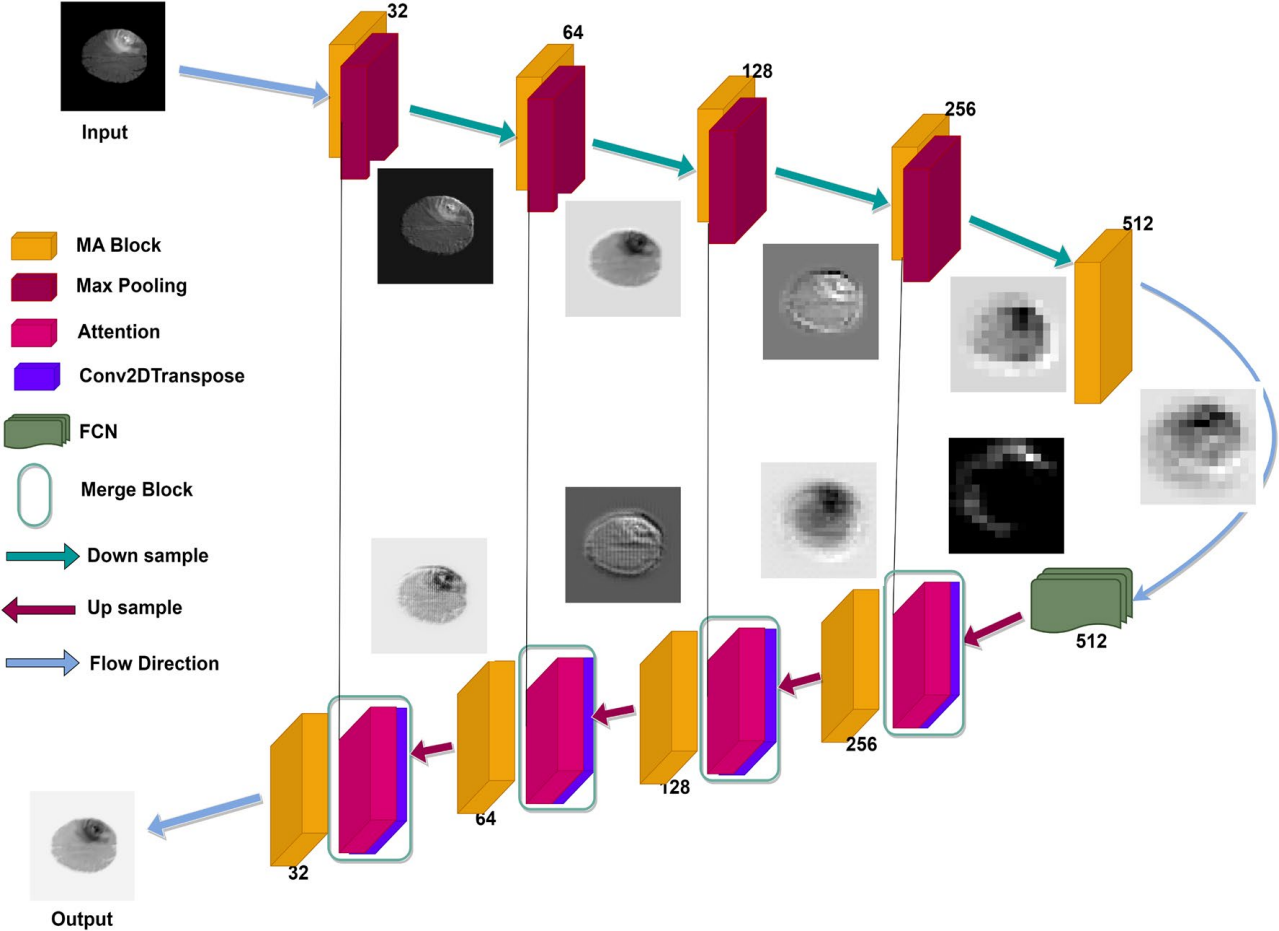
The workflow block diagram of the IC-Net architecture model.

**Figure 3:**
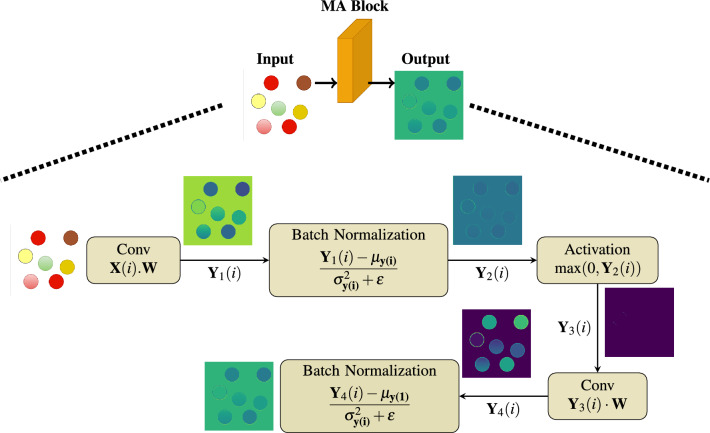
This detailed image provides an operational representation of the MA Block. Input image, featuring a composed of seven distinct colors.

The image segmentation operation of the IC-Net architecture is discussed in detail here (Fig. 2). A sample image of seven dots is taken for illustration. The image proceeds through a well-planned series of steps. The architecture starts with an input layer that accepts an image of specific dimensions followed by a five-block encoder component. An encoder block contains convolutional layers and max-pooling operations so that en Block (1 to 4) are capable of extracting hierarchical features from the input images. These convolutional layers capture complex features as we go deeper into the network, while the fifth encoder block represents the highest level of feature refinement. The operational representation of the MA Block is shown in Fig. 3. The cap-shaped structure above the block diagram, consisting of dotted lines connecting the input, MA Block, and output images, provides an overview of the flow block diagram’s processes. It processes the input image with seven colored circles through operations, starting with a convolutional layer for spatial features, followed by batch normalization for stable training and ReLU activation. A second convolutional layer refines these features, and combined, these operations enhance the image’s representation for segmentation. While the block doesn’t directly alter the output image, it primes it for segmentation. Subsequent layers in the neural network use these features for circle boundary and characteristic distinction, making convolutional layers vital for edge and pattern detection, while batch normalization and activation functions aid efficient feature learning.

**Figure 4:**
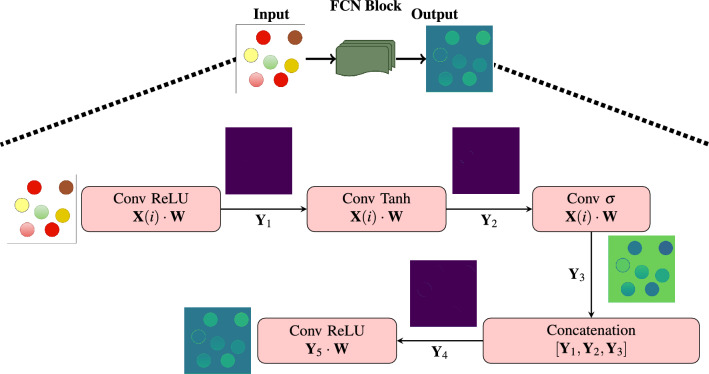
This detailed image provides an operational representation of the FCN Block. Input image, featuring a composed of seven distinct colors.

The improvement in feature representation is achieved by the Fully Convolutional Network (FCN), whose operational mechanism is shown in Fig. 4. The cap-shaped structure above the block diagram, with dotted lines connecting the input, FCN Block, and output images, provides a comprehensive overview of the flow block diagram’s operations. It systematically processes an input image. It starts with a convolutional layer using ReLU activation for spatial feature enhancement. Two additional convolutional layers follow, each employing distinct activations: tanh emphasizes intensity variations, and sigmoid highlights pixel relationships. Their outputs are concatenated to form a three-channel feature map, capturing various image aspects. A final convolutional layer with ReLU further consolidates these features. Output image remains unaltered, but this block creates a rich and diverse feature representation, ideal for segmentation. The combination of activations and concatenation allows the network to capture spatial structures, intensity variations, and pixel relationships, aiding segmentation by providing an informative and discriminative feature map.

**Figure 5:**
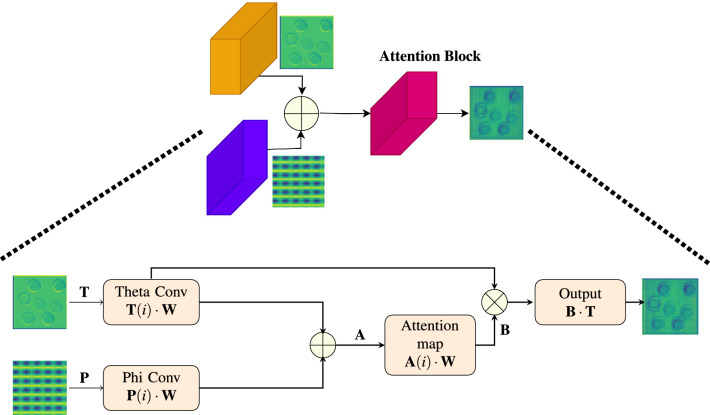
This detailed image provides an operational representation of the Attention Block.

Followed by the operation of FCN, The cap-shaped structure above the Fig. 5, with dotted lines connecting the input from the convolution block which is given to up sampling, then given to attention and output images, provides a concise summary of the processes involved. The decoder part which consists of four blocks, each with a transpose convolutional layer, an attention mechanism, and a concatenation operation comes into play. The transpose convolutional layers are essential for boosting the spatial resolution of the feature maps, and attention algorithms make sure that upsampling is targeted and context-sensitive which refers to the ability of a model to adapt its processing based on the context or surroundings of the input data. This process involves: computing “theta” and “phi” features, combining them through ReLU activation to emphasize positive relationships, calculating attention weights with a sigmoid activation (ranging from 0 to 1), and multiplying these weights with the original image. This method enhances feature representation by emphasizing regions of interest, vital for accurate segmentation. It allows the network to focus on critical areas, aiding in distinguishing object boundaries from the background and ultimately enhancing segmentation accuracy. The fifth decoder block produces segmentation map and provide class probabilities for each pixel with the help of $1\times 1$ convolutional layer and SoftMax activation. This convoluted process reaches its culmination and the operation of Attention Block is shown in Fig. 5. This analysis aids a profound understanding and a granular insight of the IC-Net’s image segmentation functionality, workflow and its performance. Overall, this methodology facilitates image segmentation through feature extraction and its refinement to produce pixel-wise class predictions in the output segmentation map. The entire operation is illustrated in Algorithm-1.

**Algorithm 1:**
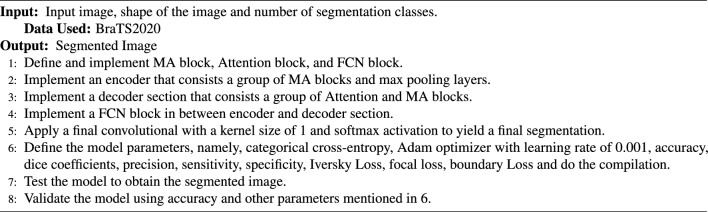
Algorithm for IC-Net Model.

Proposed methodology aims to enhance BTS accuracy by integrating MA-Blocks, FCN Block, and an attention mechanism within the IC-Net architecture as shown in Fig. 2.

We revisit the operations of MA block, FCN block and attention block functions with complete mathematical representation as shown below.

### Convolution operation

The convolution operation given an input image ${\textbf {X}}(i)$ is represented as

1 $$\begin{aligned} \quad \textbf{Y}_1(i) = \textbf{X}(i) \cdot \textbf{W} \;\; ;\; \forall i = 1,2,\cdots , v_1\cdot v_2 \end{aligned}$$

where $v_1 = v_2 = n+2p-f+1\; \;;$, ${\textbf {W}}$ is a kernel, *n*, size of input image, *p* is padding size and *f* is the filter size and $v_1$, $v_2$ are the height and width of the output feature matrix respectively. The functionalities of each blocks are explained below with the corresponding mathematical descriptions. For the easiness of illustration, we drop the subscripts in the notation and it is assumed that the input image to the architecture of each block is assumed as ${\textbf {X}}$ output as ${\textbf {Y}}$ and the outputs of intermediate stages as ${\textbf {Y}}_1$, ${\textbf {Y}}_2$, ${\textbf {Y}}_3$ and so on respectively.

- **MA Block** The operation of MA block includes convolution between ${\textbf {X}}(i)$ and ${\textbf {W}}$ to give ${\textbf {Y}}_1$ as mentioned in (1) followed by a batch normalization, $\quad \textbf{Y}_{2}(i) = \frac{\textbf{Y}_{1}(i) - {\mu }_{{\textbf {Y}}_1}}{{\sqrt{{\sigma }^2 _{{\textbf {Y}}_i} + \epsilon }}}$, $\epsilon$ is a very small positive number. Following this operation, there will be a ReLU activation ${\textbf {Y}}_3(i) = \max \left( 0, {\textbf {Y}}_2 (i) \right)$ and convolution ${\textbf {Y}}_4(i) = {\textbf {Y}}_3(i) \cdot {\textbf {W}}$ all in sequence. In these expressions, ${\textbf {X}}$ denotes input feature matrix, ${\textbf {W}}$ represent the filter used in the convolution, ${\textbf {Y}}_4$ denotes output image and ${\textbf {Y}}_1$, ${\textbf {Y}}_2$, & ${\textbf {Y}}_3$ denote intermediate output features respectively.
- **FCN Block** The operation of the FCN block comprises several key steps. It starts with a convolution operation as mentioned in (1) followed by a $\text {ReLU}(x) = \max \left( 0,x\right)$ activation function to give an intermediate output as ${\textbf {Y}}_1$. The output ${\textbf {Y}}_1$ undergoes one more convolution as in (1) followed by a Tanh Activation to give a response ${\textbf {Y}}_2$. In addition, one more convolution along with $\tanh$ is used in the next stage to give ${\textbf {Y}}_3$. It is to be noted that $\tanh = \frac{1 - e^{-2x}}{1 + e^{-2x}}$ and $\sigma (z) = \frac{1}{1 + e^{-z}}$. After convolution with Tanh Activation, another convolution between ${\textbf {Y}}_2$ and ${\textbf {W}}$ as in (1) followed by a $\sigma$ activation function is applied to get ${\textbf {Y}}_3$. Now all these are concatenated using $\textbf{Y}_4 = \mathbf {Y_1} || \mathbf {Y_2} || \mathbf {Y_3}$, where || denotes column wise concatenation. Final response ${\textbf {Y}}$ is obtained by doing another convolution between $\mathbf {Y_4}$ and ${\textbf {W}}$. In these equations, $\textbf{X}$ and $\textbf{Y}$ represents the input and output feature matrix respectively. $\textbf{Y}_1$, $\textbf{Y}_2$, $\textbf{Y}_3$ and $\textbf{Y}_4$ represent output after convolution with Tanh, $\sigma$ and ReLU respectively. $\textbf{Y}_4$ denoted concatenated feature matrix.
- **Attention Block** It encompasses several integral components and operations. We have the input feature matrix $\textbf{T}{(i)}$, along with an additional input feature matrix $\textbf{P}{(i)}$. Output feature matrix $\textbf{B}$ is the result of the convolution operation, while the application of attention weights yields the output feature matrix $\textbf{Y}$. In this Operations, Firstly theta convolution operation $\textbf{T} = \textbf{T}(i) \cdot \textbf{W}$ takes place as in (1). Similar operation takes place to operate on $\textbf{P}$ in Phi convolution. These results are added together to yield $\textbf{A}$, with a subsequent application of the ReLU activation function. Output from the addition is processed further through the operation $\textbf{B} = \textbf{A}(i) \cdot \textbf{W}$ as in (1). Finally, the output with attention is obtained as $\textbf{Y} = \textbf{B} \cdot \textbf{T}$.

Firstly, MA-Blocks inspired by recent advancements in attention mechanisms are integrated into IC-Net to focus on pertinent tumor regions within the input, incorporating both global and local context information. The MA-Block function, a vital component of IC-Net, is detailed, encompassing specific operations such as a $3\times 3$ convolutional layer, batch normalization, ReLU activation, and another convolutional layer. The architecture of these MA-blocks allows for adaptive feature map weighting, effectively capturing intricate patterns within the data. It improves spatial integrity and capturing delicate features for subsequent layers. Also, ensures that it identifies detailed patterns in input data using convolutional layers, batch normalization, and ReLU activation, improving spatial integrity and capturing delicate features for subsequent layers. These blocks play a pivotal role in the encoder section, aiding in the extraction of hierarchical features from the input image across various scales through progressive down-sampling via max-pooling layers. The number of filters employed in each convolutional block is indicated by the numerals (32, 64, 128, 256, 512). Furthermore, FCN are integrated within IC-Net to capture multi-scale information and enhance the network’s capability to handle tumors of varying sizes and shapes. The FCN blocks are thoroughly explained, emphasizing the concatenation of features derived from various activation functions (ReLU, tanh sigmoid), significantly enriching feature representations by effectively combining information from multiple activation functions. It also enhances the network’s feature capture by utilizing various activation functions and encouraging multi-scale feature extraction. Transforms unstructured data into a multimodal feature map with various semantic characteristics. Achieves convergence while maintaining spatial coherence using convolutional techniques and concatenation.

**Table 3:** Hyperparameters used for training the network.

Hyperparameters	Description
Loss function	Categorical Crossentropy
Optimizer and Learning rate	Adam (learning rate=0.001)
Metrics	(Accuracy, dice_coef, precision, sensitivity, specificity, Tversky loss, focal loss, boundary loss)

An attention mechanism is used to emphasize critical spatial information between two feature maps, focusing on relevant parts of the input image while suppressing irrelevant information consequently enhancing segmentation accuracy. This mechanism, strategically placed within the decoder part of the model, enhances the network’s ability to focus on critical regions. The mechanism involves specific steps such as 1x1 convolutions, element-wise addition, ReLU activation, dimensionality reduction, and the creation of an attention mask. Organizes an elegant attention performance by incorporating input feature maps and contextual data. It directs the network’s attention, improving its interpretive abilities. Provides neural networks with specific focus capabilities. Perpetually allocates attention to regions within the input space, enhancing interpretative prowess and predictive accuracy. The integration of these components, MA-Blocks, FCN blocks, and the attention mechanism, forms a robust methodology for BTS. The model’s segmentation accuracy is enhanced by utilizing attention mechanisms, diverse activation functions, and adaptive feature weighting to enhance feature representation.

Impact of IC NET - Our IC-Net framework presents a number of novel developments, each of which adds in a different way to its improved usefulness and speed. Our architecture’s MA block uses a simple yet efficient series of convolutional, batch normalization, and activation layers to facilitate deeper network representation while streamlining feature extraction. Our FCN block, on the other hand, is unique in that it uses parallel convolutional layers with several activation functions (ReLU, tanh, and sigmoid) to enable the combination of multiple feature representations in a single block. In the meantime, the attention block integrates a complex channel attention mechanism that enables the model to selectively emphasize important characteristics across channels. It does this by using gating signals and convolutions on input feature maps to build attention maps. Our architecture has been shown superior through extensive testing and comparative analyses, which reveal its higher performance in tasks like classification and segmentation on benchmark datasets. Feature map and attention mechanism visualizations demonstrate how well our architecture can capture complex patterns and highlight important regions, while thoughtful design decisions in each block address particular shortcomings in traditional systems. These developments, in conjunction with use case cases in highly specialized areas such as remote sensing and medical picture segmentation, establish our IC-Net as a cutting edge and very efficient solution for image analysis work.

## Results and discussion

For the implementation, we utilized PyCharm as our development environment. Our system configuration was centered around a PowerEdge R740 server equipped with an INTEL XEON Silver 4208 2.1 GHz processor, a Tesla V100 GPU boasting 8 cores and 16 threads, delivering 9.6 gigapixels per second (GP/s) of graphics processing power, and backed by 128 GB of DDR4 RAM. We import TensorFlow, a deep learning framework, and Keras, a high-level API for model construction. It includes layers like dense, convolutional, and recurrent, and the Model class for defining and configuring deep learning models. It serves as a foundational step for building and training neural networks for various machine learning tasks. According to the experimental findings, IC-Net performs better at tumor segmentation than conventional U-Net models. Utilizing criteria like accuracy, sensitivity, specificity, and precision, the model’s performance is assessed. To establish the optimal segmentation performance, it is trained on the brain MRI dataset BraTS2020. During our experimental time, we encountered constraints, choosing an optimizer and computational resources. Switching to GPU-based training was necessary for increased effectiveness. The Adam optimizer was chosen due to flexibility. Addressing hyperparameter alteration, data constraints, and model complexity is crucial.

**Figure 6:**
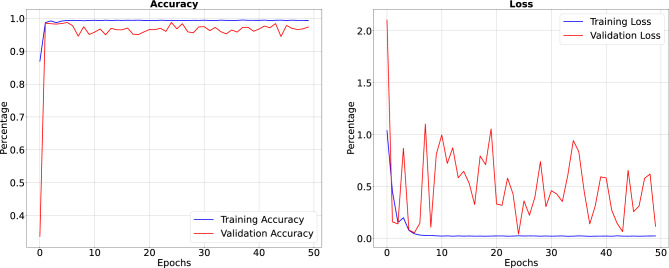
Accuracy and loss when trained for 50 Epoch.

**Figure 7:**
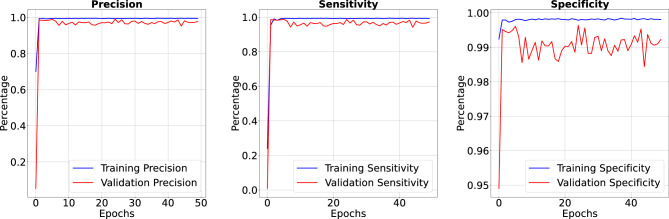
Precision, sensitivity and specificity when trained for 50 Epoch.

**Figure 8:**
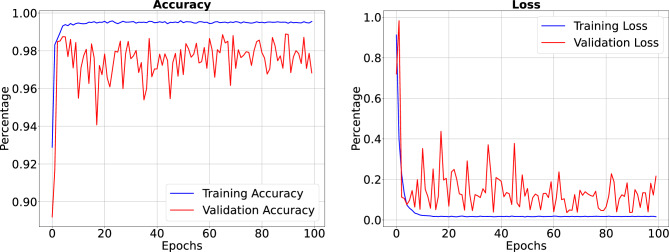
Our model achieved 99.6% accuracy in 100 Epoch training, despite a cluttered visual representation due to the intricate level of detail beyond the 99. Model’s loss value of 0.0159 resulted in a cluttered appearance due to the intricate level of detail.

**Figure 9:**
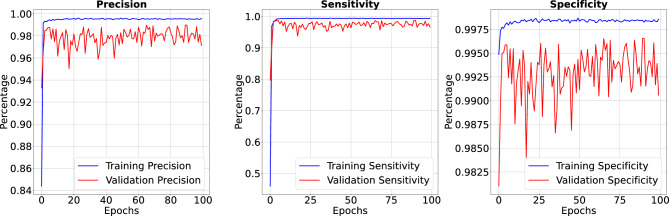
Precision, sensitivity and specificity when trained for 100 Epoch. Our IC-Net has achieved high precision, sensitivity, and specificity values, resulting in a detailed presentation with performance metrics beyond typical ranges.

### Model training parameters training approach

Using categorized training data we trained our model using the Adam optimizer with a learning rate of 0.001 and the loss function as Categorical Crossentropy. Measures used to calculate the performance of the model includes: Accuracy, Precision, Sensitivity, Specificity, DSC (core, whole, and enhancing tumors), Tversky, Focal and boundary loss. Important detailing of the dataset used have been discussed above in section "Dataset" and Important basics of our technique on preprocessing processes to the dataset in section "Pre-processing". These details were vital for guaranteeing the repeatability of our findings and enabling a thorough evaluation of our IC-Net methodology. With four different MRI sequences, the model suggested in this research is separately trained and validated four times. Hyper-parameters used for training the network are described in Table 3. The number of floating-point operations (FLOPs) for a convolutional layer is calculated as twice the product of the number of kernels, the kernel height, the kernel width, the output height, and the output width. Number of FLOPs for a fully connected layer is calculated as twice the product of the input size and the output size. Using the above details, the total FLOPs for the MA Block can be calculated by multiplying twice the kernel size by the input channels, output channels, height, and width. For the Attention Block, the total FLOPs involve the sum of two sets of operations: the convolutional layers and the subsequent addition, activation, and multiplication operations. The total FLOPs for the FCN (Fully Convolutional Network) Block are determined by tripling twice the kernel size and multiplying by the input channels, output channels, height, and width. With the above reference and given assumptions, the FLOPs for the MA Block, Attention Block, and FCN Block were calculated to be about 2,147,483,648 FLOPs, 3,221,225,472 FLOPs, and 4,294,967,296 FLOPs, respectively. Total FLOPs for the model were determined to be around 9,663,676,416 FLOPs. Our system requirement me have mentioned it in section "Results and discussion" results and discussion. The FLOPs per second for our Tesla V100 GPU is rounded $9.66 \, \text {GFLOPs/s}$ (gigaflops per second). Time consumption can be calculated when Number of Training Samples is multiplied with number of Epochs and time per epoch and when divided with number of GPU cores will give the training time. The assessed training period, calculated based on the number of GPU cores, number of training samples, number of epochs and the time per epoch, is about 3112.5 s. For calculating memory consumption be Trainable params which can be added with non-trainable params multiplied by size of parameter in bytes. Our IC-Net memory consumption is estimated based on the sum of trainable and non-trainable parameters; it approximately sums to 63.23 megabytes.

**Table 4:** On the BraTS2020 dataset, a comparison of IC-Net with state-of-the-art techniques.

Model	Accuracy	Loss	Precision	Sensitivity	Specificity
Modified U-Net structure^37^	0.934	0.26		0.857	0.917
U-net with ResNet34^27^	0.88	0.20	0.792		
U-Net with VGG-19^44^	0.991	0.054	0.993	0.98	0.981
Hybrid DL^45^	0.967		0.980	0.980	0.963
Improved U-Net^46^	0.9671		0.9806	0.9806	0.9833
U-Net3+ with Stage Residual^47^	0.971			0.98	0.963
SLf-UNet^48^	0.95	0.05	0.92	0.90	0.94
Two-pathway residual Encoder Decoder-UNet (TPRED-UNet)^49^	0.9973	0.0031		0.8919	0.9987
TPRD-UNet^49^	0.9972	0.0027		0.8831	0.9987
Znet^50^	0.9955	0.0839			
Deep CNN and U-Net^51^	0.987	0.066	0.984	0.987	0.996
Our IC-Net	0.9965	0.0159	0.9958	0.9944	0.9986

**Table 5:** Performance of our IC-Net.

Performance matrix	Training of IC-net	Val of IC-net
Accuracy	99.65	97.94
Loss	0.0159	0.1293
Precision	99.58	98.69
Sensitivity	99.44	98.75
Specificity	99.86	99.42
F1 Score	98.66	96.87
Focal loss	3.4754	3.5650
Tversky loss	0.0095	0.0274
Dice coef edema	0.7439	0.5459
Dice coef enhancing	0.7551	0.5686
Boundary loss	0.0216	0.0432

**Figure 10:**
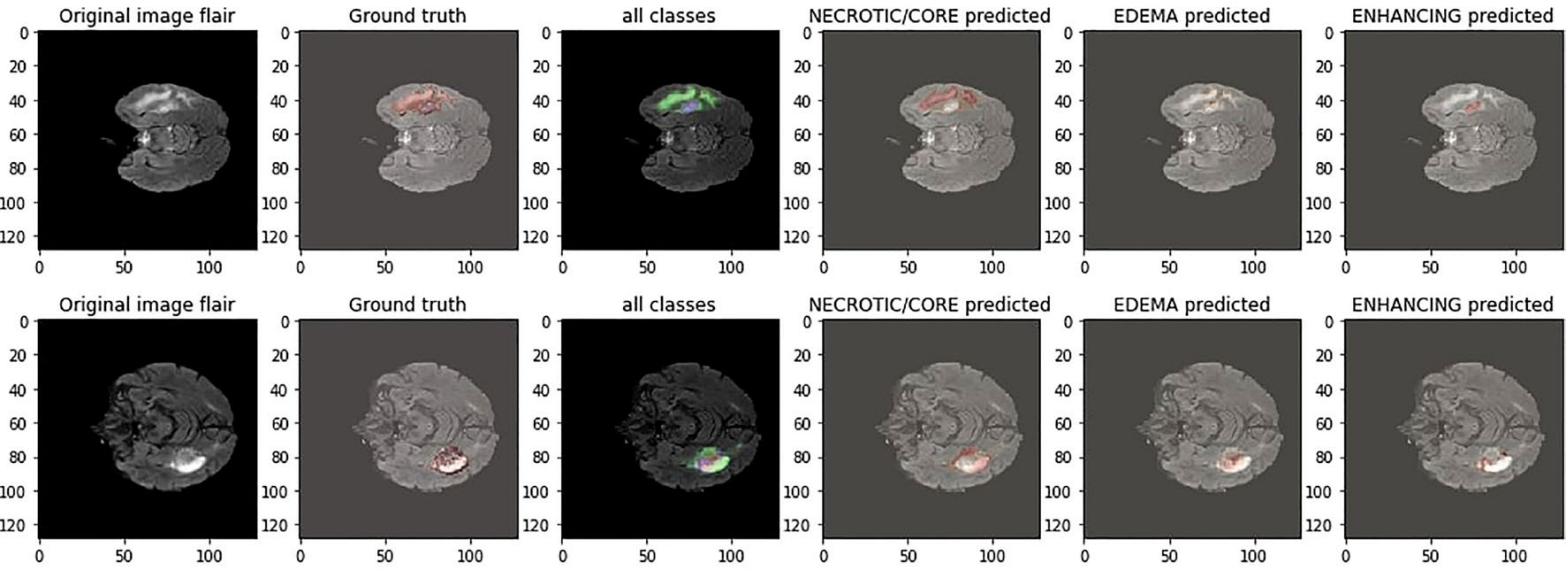
This image displays existing results, serving as a point of comparison with the visualization of qualitative results on BraTS2020 MRI sequences in^43^.

**Figure 11:**
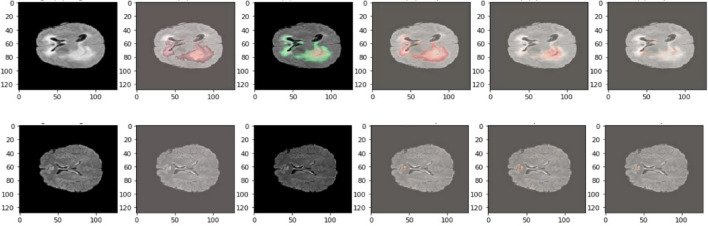
This image displays existing results, serving as a point of comparison with the visualization of qualitative results on BraTS2020 MRI sequences in^44^.

**Figure 12:**
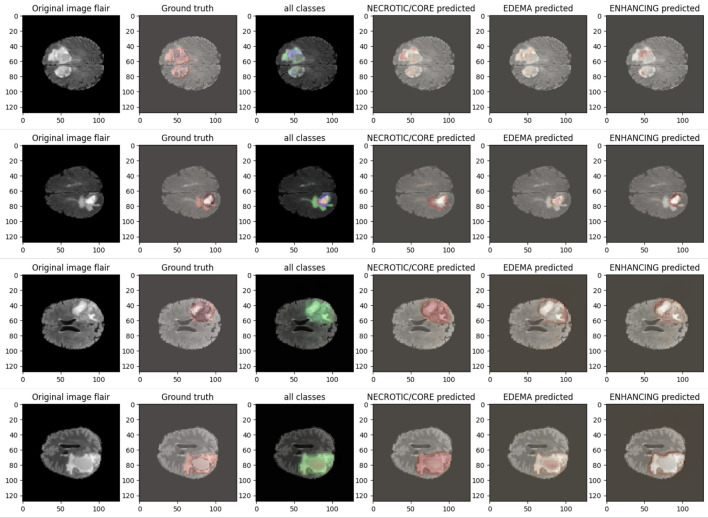
This image displays visualization of qualitative results on BraTS2021.

**Figure 13:**
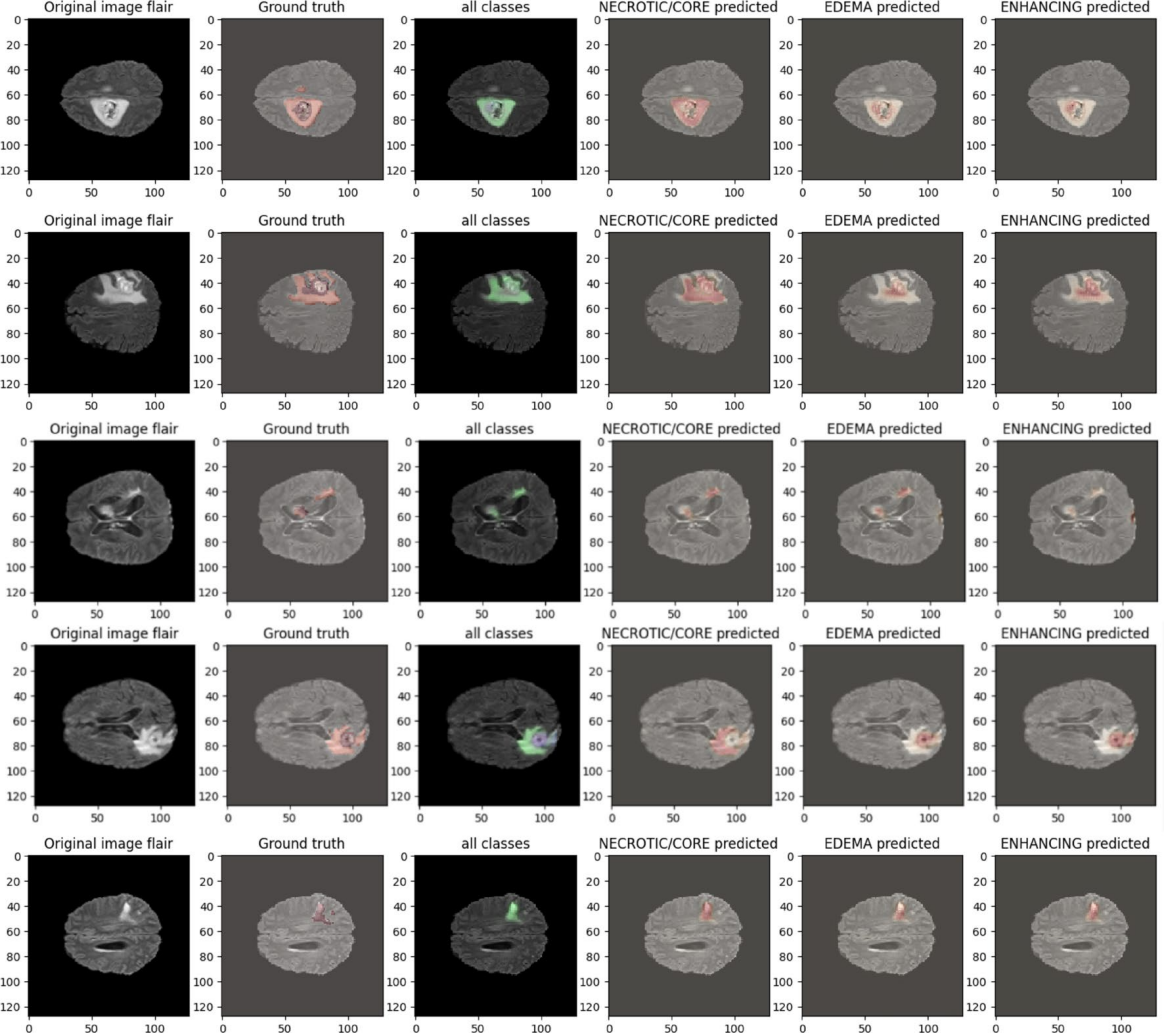
After training the model, the following visualization showcases the qualitative results of the IC-Net model on BraTS2020 MRI sequences.

**Figure 14:**
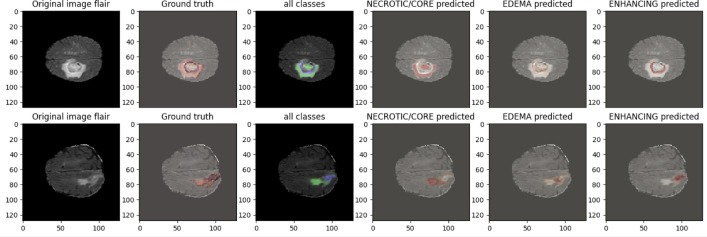
After training the model, the following visualization showcases the qualitative results of the IC-Net model on BraTS2019 MRI sequences.

**Table 6:** Performance comparisons of subclass tumor using Brats2020 data.

Algorithms	DSC (WT)	DSC (TC)	DSC (ET)
Self-calibrated attention U-Net^50^	0.905	0.821	0.781
Double attention U-Net^33^	0.8912	0.8427	0.7915
AD-Net^22^	0.872	0.823	0.803
dResU-Net^24^	0.8660	0.8004	0.8357
Attention-based CNN with U-Net^44^	0.90	0.86	0.83
Aggregation-and-Attention Network^52^	0.93	0.88	0.87
Znet^50^	0.839	0.762	0.746
Automated Multimodal^53^	0.840	0.780	0.760
Deep multi-task learning with multi-depth fusion^54^	0.860	0.772	0.700
Convolutional block attention - V-Net^55^	0.876	0.769	0.670
AGSE-VNet^56^	0.68	0.85	0.70
IC-Net	0.998717	0.888930	0.866183

**Table 7:** Performance comparisons of subclass tumor using Brats2021 data.

Algorithm	WT	TC	ET
dResU-Net^24^	0.8601	0.8400	0.8221
Multi-scale spatial distillation pseudo-labeling^57^	0.925	0.890	0.876
Adversarial learning^58^	0.9077	0.8539	0.8138
U-Net with Attention block^59^	0.879	0.819	0.793
Bridged U-Net_ASPP (var-1)^60^	0.9187	0.8594	0.8434
Bridged U-Net_ASPP (var-2)^60^	0.9251	0.8658	0.8395
IC-Net	0.9977	0.8594	0.8526

**Table 8:** Performance comparisons of subclass tumor using Brats2019 data.

Algorithm	WT	TC	ET
SCAU-Net^32^	0.907	0.820	0.782
Multi-step Cascaded Networks^61^	0.886	0.813	0.771
MECU-Net^62^	0.902	0.824	0.777
TransConver U-Net^63^	0.825	0.901	0.784
AugTransU-Net^64^	0.897	0.782	0.804
IC-Net	0.9941	0.8462	0.8278

**Figure 15:**
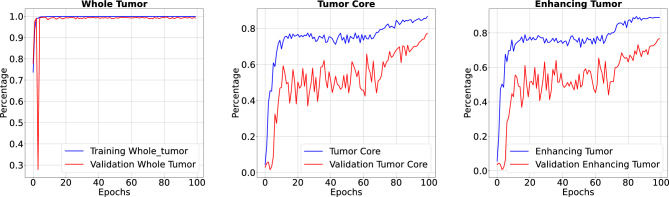
Following visualization shows the subclass results of the IC-Net model on BraTS2020 MRI Data.

**Figure 16:**
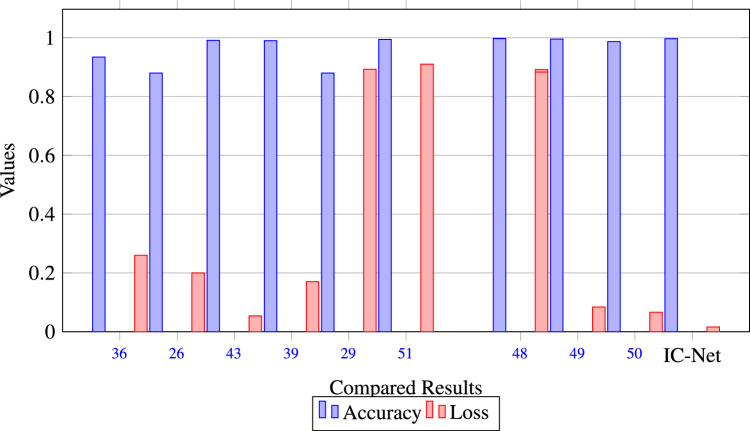
Comparison results of Our Model compared with existing methods.

### Evaluation metrics

Accuracy, sensitivity, precision and specificity are used to evaluate the model based on the provided segmented ground truth of the tumor part in the MRI are then calculated using following Eqs. (2), (3), (4), (6) and (7). By dividing the total number of predictions by the number of right predictions, accuracy processes determine how exact each prediction is. The precision technique highlights exactness by calculating the percentage of true positive predictions among all positive forecasts. Remember processes the percentage of all real positive cases that are accurate positive predictions, with an emphasis on completeness.

2 $$\begin{aligned} \text {Accuracy}= & {} \frac{TP+TN}{TP+TN+FP+FN} \end{aligned}$$

3 $$\begin{aligned} \text {Precision}= & {} \frac{TP}{TP+FP} \end{aligned}$$

4 $$\begin{aligned} \text {Recall}= & {} \frac{TP}{TP+FN} \end{aligned}$$

DSC score performance metric computes the similarity percentage between the ground truth and the output of a model. Suppose, *C* and *D* are two sets, the dice similarity of these two sets are then calculated with Eq. (5)

5 $$\begin{aligned} \text {DSC} = \frac{2*|C \cap D|}{|C|+|D|} \end{aligned}$$

Sensitivity is calculated with Eq. (6) where, cardinalities of sets *C* and *D* are denoted with |*C*| and |*D*| respectively, $G_1$ representing the proportion of tumor regions of ground truth images and $C_1$ represents tumor regions that were predicted by the model.

6 $$\begin{aligned} \text {Sensitivity}(C,G)=\frac{|C_1 \wedge G_1|}{|G_1|} \end{aligned}$$

Specificity is calculated with Eq. (7) where $G_0$ represents non-tumor tissue regions of the ground truth and $C_0$ represents the non-tumor tissue regions predicted by the model.

7 $$\begin{aligned} \text {Specificity}(C,G)=\frac{|C_0 \wedge G_0|}{|G_0|} \end{aligned}$$

### Model evaluation

In the results section of our research paper, we are delighted to report exceptionally high-performance metrics obtained after training our model for 50 and 100 epochs, underscoring the effectiveness of our approach. For the 50-epoch experiment, we achieved accuracy rates consistently exceeding 99.39%, coupled with low loss value of 0.0246, indicating the model’s strong predictive capabilities and the minimization of errors. Precision scores consistently above 99.43% demonstrate the model’s proficiency in correctly identifying positive cases while keeping false positives to a minimum. Sensitivity consistently exceeding 99.26%. This indicates the model’s ability to successfully capture a significant proportion of true positive cases. Specificity above 99.79%, reflecting its capability to effectively distinguish between negative and positive instances. Results for 50 epoch are displayed in Figs. 6 and 7. For the 100-epoch experiment, our model consistently achieved accuracy rates exceeding 99.65%, maintained low loss values of 0.0159, and demonstrated precision scores of 99.58%, minimizing false positives. Moreover, our research exhibited high sensitivity exceeding 99.44%, indicating the model’s ability to capture true positive cases, and specificity consistently of 99.86%, highlighting its proficiency in distinguishing negative instances. Remarkably, these high-performance metrics remained stable and even improved marginally in the 100-epoch experiment, demonstrating the robustness and reliability of our methodology over extended training periods are given in Figs. 8 and 9. Our model consistently outperforms advanced methods in performance metrics, demonstrating its superiority in various aspects. Key findings summarize these results in Table 4 and results when compared with existing methods are given in Fig. 16 in a graphical way. To make sure that our results were reliable and consistent, we ran the model several times during our studies. These numerous runs yielded the following accuracy values: 099636, 099635, 099664, 099667, 099635, 099664, 099667, and 099635. We determined the standard deviation of these accuracy numbers in order to further verify the stability of our framework. Performance of our model displays a high degree of consistency and reliability, as evidenced by the standard deviation of 0.00014. This low standard deviation supports the effectiveness of our method by showing that the accuracy of our model is both high and consistent across runs.

Our research demonstrated the effectiveness of our approach, with high accuracy, low loss, and notable precision, sensitivity, and specificity scores, indicating its reliability and potential contributions. Performance of our IC-Net resuts are presented in Table 5. The results provide a solid foundation for our research paper’s conclusions, demonstrating the effectiveness of our methodology in achieving our intended goals.

Our analysis reveals that our approach outperforms^43,44^ in the segmentation of brain tumors using the BraTS2020. Figures 10 and 11 represent the prediction using existing models which outperforms by our IC-Net. Figures 12 and 14 shows the segmentation result of brain tumors using Brats 2021 and 2019 data (Figs. 13, 14). The superior results are evident in the accuracy of delineating tumor classes and individual core, whole, and enhancing tumors, showcased in columns four, five, and six of the images. Tables 6, 7 and 8 provides a comparison of the DSC values of 2020, 2021 and 2019 brats data for Whole Tumor (WT), Tumor Core (TC), and Enhanced Tumor (ET) with the most recent algorithms. In addition, the graphical depiction of the outcomes for WT, TC, and ET is shown in Fig. 15. Results of our IC-Net shows promising visuals which are presented below in Fig. 13. Our model effectively defines data boundaries, improving segmentation quality and precision. It yields more accurate, visually appealing results, highlighting its practical value in real-world applications for improved decision-making processes for clinical applications and medical image analysis (Fig. 16).

## Conclusion

In summary, IC-Net is a novel semantic segmentation architecture that combines MA-blocks, FCN, attention blocks and an encoder-decoder structure. It achieves state-of-the-art results in segmentation tasks by effectively capturing complex features and context information. Our manuscript provides a comprehensive explanation of the IC-Net framework and its constituent components, along with experimental results demonstrating its effectiveness. Our results of tumor segmentation are influenced differently by many variables. This research suggests a multimodal BTS method based on IC-Net to make better use of multimodal brain tumor image data. Through wide experimentations and quantitative evaluations, we validate the superiority of our IC-Net model compared to other state-of-the-art segmentation methods. Our proposed modifications have proven highly accurate and reliable in achieving BTSs, even in challenging scenarios with irregular tumor shapes and variable appearances.

## Future directions

We outline several paths for future research, including model refinement, domain adaptation, and real-time clinical deployment. Outline the development and evaluation of IC-Net, a modified neural network model for BTS. Through rigorous experiments, we demonstrate that IC-Net offers superior performance compared to traditional U-Net and other existing models. Subsequent investigations will concentrate on incorporating IC-Net into clinical procedures, guaranteeing its usability and efficacy in practical contexts. The model will be created with ease of integration into current systems, enabling real-time tumor segmentation and supporting the choice of diagnosis and course of treatment. Performance evaluations will take place in actual clinical settings, and input from medical professionals will be vital to improving the model. Other medical imaging tasks will also be added to IC-Net, which could enhance patient outcomes and diagnostic precision. To improve IC-Net’s usefulness in medical imaging, future studies should examine how it may be integrated into clinical workflows, evaluate how well it works in practical situations, and extend its use to additional medical imaging modalities like PET, CT, and ultrasonography images. Our research contributes to the ongoing efforts to enhance medical image analysis, aiming to improve patient care and treatment outcomes in neuro-oncology.

## Data Availability

The dataset was gathered from the Brain Tumor Segmentation 2020 Dataset, which is available online. It is publicly accessible and unrestricted. https://www.kaggle.com/awsaf49/brats20-dataset-training-validation.
